# Anandamide Capacitates Bull Spermatozoa through CB1 and TRPV1 Activation

**DOI:** 10.1371/journal.pone.0016993

**Published:** 2011-02-11

**Authors:** María Gracia Gervasi, Claudia Osycka-Salut, Julieta Caballero, Mónica Vazquez-Levin, Elba Pereyra, Silvia Billi, Ana Franchi, Silvina Perez-Martinez

**Affiliations:** 1 Centro de Estudios Farmacológicos y Botánicos, Facultad de Medicina, Consejo Nacional de Investigaciones Científicas y Técnicas-Universidad de Buenos Aires, Buenos Aires, Argentina; 2 Instituto de Biología y Medicina Experimental-Consejo Nacional de Investigaciones Científicas y Técnicas, Buenos Aires, Argentina; 3 Instituto de Investigaciones Biotecnológicas, Universidad de San Martín, Buenos Aires, Argentina; State Key Laboratory of Reproductive Biology, Institute of Zoology, Chinese Academy of Sciences, China

## Abstract

Anandamide (AEA), a major endocannabinoid, binds to cannabinoid and vanilloid receptors (CB1, CB2 and TRPV1) and affects many reproductive functions. Nanomolar levels of anandamide are found in reproductive fluids including mid-cycle oviductal fluid. Previously, we found that R(+)-methanandamide, an anandamide analogue, induces sperm releasing from bovine oviductal epithelium and the CB1 antagonist, SR141716A, reversed this effect. Since sperm detachment may be due to surface remodeling brought about by capacitation, the aim of this paper was to investigate whether anandamide at physiological concentrations could act as a capacitating agent in bull spermatozoa. We demonstrated that at nanomolar concentrations R(+)-methanandamide or anandamide induced bull sperm capacitation, whereas SR141716A and capsazepine (a TRPV1 antagonist) inhibited this induction. Previous studies indicate that mammalian spermatozoa possess the enzymatic machinery to produce and degrade their own AEA via the actions of the AEA-synthesizing phospholipase D and the fatty acid amide hydrolase (FAAH) respectively. Our results indicated that, URB597, a potent inhibitor of the FAAH, produced effects on bovine sperm capacitation similar to those elicited by exogenous AEA suggesting that this process is normally regulated by an endogenous tone. We also investigated whether anandamide is involved in bovine heparin-capacitated spermatozoa, since heparin is a known capacitating agent of bovine sperm. When the spermatozoa were incubated in the presence of R(+)-methanandamide and heparin, the percentage of capacitated spermatozoa was similar to that in the presence of R(+)-methanandamide alone. The pre-incubation with CB1 or TRPV1 antagonists inhibited heparin-induced sperm capacitation; moreover the activity of FAAH was 30% lower in heparin-capacitated spermatozoa as compared to control conditions. This suggests that heparin may increase endogenous anandamide levels. Our findings indicate that anandamide induces sperm capacitation through the activation of CB1 and TRPV1 receptors and could be involved in the same molecular pathway as heparin in bovines.

## Introduction

Mammalian spermatozoa are not able to fertilize an egg immediately upon ejaculation. They acquire this ability during their transit through the female genital tract in a process known as capacitation, where they undergo a large number of membrane and metabolic modifications such as an increase in intracellular ions and protein tyrosine phosphorylation, generation of reactive oxygen species and changes in metabolism, motility and plasma membrane fluidity [1]–[5].

The mammalian oviduct acts as a functional sperm reservoir providing a suitable environment that allows the maintenance of sperm fertilization competence until ovulation occurs [6]. The interaction between oviductal epithelial cells and spermatozoa is thought to prolong sperm life by delaying capacitation until ovulation-associated signals [7], induce the release of adhering sperm subpopulations [7]–[8]. Conditioned media from whole oviduct [9] or monolayers of oviductal epithelial cells [10]–[11] have a capacitating activity that peaks at estrous and declines during the luteal phase, suggesting that some molecules contained in the oviductal fluid could act as capacitating agents. In cattle, heparin or heparin-like glycosaminoglycans present in the oviductal fluid are considered potential *in vivo* capacitating agents [12]–[15]. Indeed, bull spermatozoa are capacitated *in vitro* by exposure to different glycosaminoglycans such as heparin, hyaluronan and heparan sulphate [12], [16].

Anandamide (AEA) is an endocannabinoid that activates cannabinoid receptor 1 (CB1) and cannabinoid receptor 2 (CB2), located on the surface of target cells [17]. Anandamide may also act as an endovanilloid, through the activation of the transient receptor potential vanilloid type I (TRPV1) [18]–[19]. We have recently demonstrated that bull spermatozoa express CB1, CB2 and fatty acid amide hydrolase (FAAH), the enzyme that degrades AEA and regulates its endogenous levels. We also found that AEA, at nanomolar concentrations, promotes sperm release from bovine oviductal epithelium, by activating CB1 but not CB2, without modifying sperm motility and acrosome reaction levels [20]. Consistently, boar and human spermatozoa exhibit a completely functional endocannabinoid system related to AEA that binds (CB1 and TRPV1), synthesizes (AEA-synthesizing phospholipase D (NAPE-PLD)) and degrades (FAAH) AEA [21]–[24]. This indicates that the spermatozoa possess the enzymatic machinery to produce and degrade their own AEA exhibiting an endogenous anandamide tone. Several works indicate that cannabinoids and vanilloids receptors are involved in sperm functions. The activation of CB1 and TRPV1 modulates boar sperm function and the AEA-binding TRPV1 receptor could be involved in human sperm fertilizing ability [21]–[22]. Recent findings have demonstrated that CB1 plays a new role in the control of sperm energy homeostasis [25]. In addition, Agirregoitia *et al*. [26] have recently found that CB1 and CB2 receptors are differentially involved in regulating human sperm motility.

Nanomolar levels of AEA are found in reproductive fluids including mid-cycle oviductal fluid and follicular fluid [27]–[28]. Thus, spermatozoa are sequentially exposed to AEA as they swim from the vagina to the fertilization site in the oviductal ampulla. Studies in different mammalian spermatozoa suggest the existence of an AEA tone in the oviduct that might regulate sperm capacitation [22], [24], [29] and its fertilizing potential within the reproductive tract.

Our evidences indicate that AEA signaling might regulate sperm-oviduct interaction [20]. Since sperm detachment may be due to surface remodeling by capacitation, we therefore propose that during the peri-ovulatory period oviductal AEA induces sperm capacitation thus promoting sperm release from the oviductal reservoir towards the fertilization site. To evaluate the physiological relevance of this hypothesis it is necessary to determine whether oviductal concentrations of AEA are able to capacitate bovine spermatozoa. Therefore, the aim of this work was to investigate whether AEA at physiological concentrations could induce sperm capacitation in bull spermatozoa. The participation of CB1, CB2 and/or TRPV1 receptors was also analyzed. Finally, we investigated whether AEA and heparin share the same activation pathway.

## Results

### Effect of AEA on sperm capacitation

Sperm capacitation consists of a complex series of finely tuned events such as changes in plasma membrane fluidity, increase in sperm intracellular free Ca^2+^ concentration and the phosphorylation of protein tyrosine residues [3]. In this work, sperm capacitation related-events were evaluated by chlortetracycline (CTC) assay, induction of protein tyrosine phosphorylation patterns and L-α-lysophosphatidylcholine (LPC)-induced acrosome reaction assessed by *Pissum sativum* agglutinin-FITC staining (PSA-FITC).

**Figure 1:**
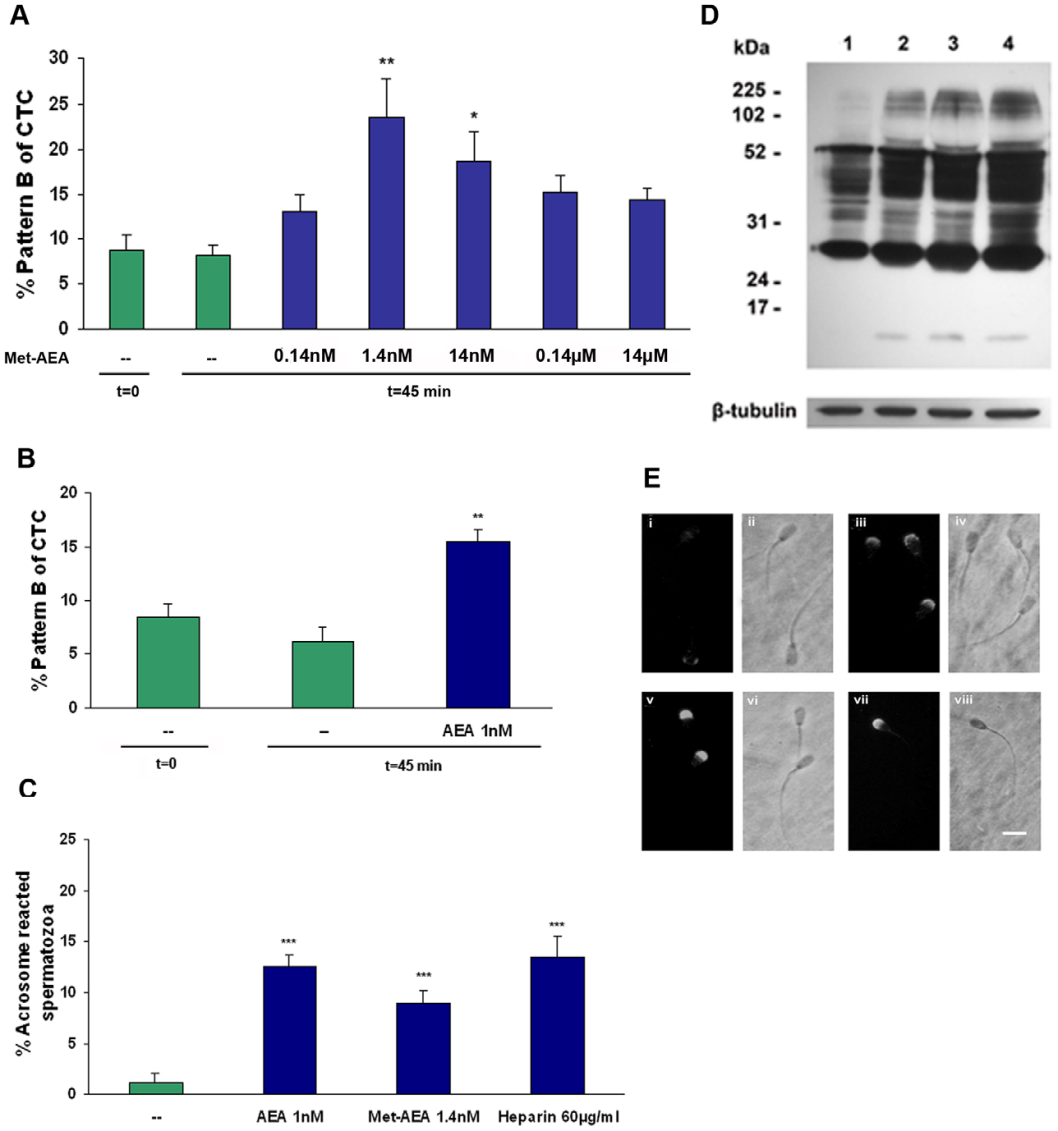
Effect of R(+)methanandamide (Met-AEA) and anandamide (AEA) on bull sperm capacitation. Spermatozoa were incubated for 45 min at 38.5°C in sp-TALP medium with Met-AEA or AEA. Bars indicate the percentage of capacitated spermatozoa (A and B: % pattern B of CTC; C: % acrosome reacted spermatozoa). Data are expressed as mean±SEM. **A, B**: Assessment of sperm capacitation by CTC assay. **A**: Effect of increased concentrations of Met-AEA (0.14 nM-1.4 µM); (—) t = 0 and t = 45, sp-TALP at 0 and 45 min (control) incubation respectively (n = 5). **B**: Effect of AEA (1 nM); t = 0 and t = 45 (controls) (n  =  6). **C**: Assessment of sperm capacitation by LPC-induced acrosome reaction (AR)-PSA-FITC. Heparin (positive control) or (—) sp-TALP (control). Spermatozoa were incubated for 45 min at 38.5°C in sp-TALP medium with Met-AEA (1.4 nM) or AEA (1 nM) and then were incubated for 15 min either with or without LPC to induce AR. Bars show percentage of spermatozoa that underwent LPC-induced AR minus the percentage of spermatozoa that underwent spontaneous AR (n = 4). **D**: Evaluation of protein tyrosine phosphorylation. Sperm proteins were extracted and subjected to SDS-PAGE immunoblotting with a specific monoclonal antibody against phosphotyrosine (clone 4G10). Antibody against β-tubulin (50 kDa) was used as loading control. A representative experiment is shown. Numbers on the left-hand side of the gel represent the position of the relative molecular mass standards. Lane 1: sp-TALP (control), lane 2: Met-AEA (1.4 nM), lane 3: Heparin (60 µg/ml), lane 4: AEA (1 nM), (n = 3). **E**: Immunolocalization of tyrosine phosphorylated-proteins in bovine spermatozoa. Panels i and iii: sp-TALP (control), panel v: Met-AEA (1.4 nM), panel vii: Heparin (60 µg/ml), panels ii, iv, vi, and viii: phase contrast (scale bar = 10 µm). Specificity of the reaction was tested omitting the first antibody (Magnification, X400). *p<0.05 vs control; **p<0.01 vs. control; ***p<0.001 vs. control.

CTC analysis provides a useful method for assessing intracellular calcium mobilisation in mammalian spermatozoa [30]. *In vitro* sperm capacitation experiments were performed with different concentrations of R(+)-methanandamide (Met-AEA), a non-hydrolysable AEA analogue [31]. Spermatozoa incubated in sp-TALP medium alone for 45 min were used for comparison. It was observed that Met-AEA promoted sperm capacitation at 1.4 and 14 nM concentrations compared to the control sample. The extent of capacitated spermatozoa is about twofold higher (23% at 1.4 nM Met-AEA concentration) compared to the control sample (8%) (Fig. 1A). Interestingly, Met-AEA, at either lower or higher concentrations, did not induce sperm capacitation (Fig. 1A). Anandamide (1 nM) also produced a significant increase in pattern B (Fig. 1B).

In bovine spermatozoa the LPC-induced acrosome reaction is considered an appropriate endpoint marker for capacitation [12]. Spermatozoa incubated with nanomolar concentrations of Met-AEA or AEA for 45 min showed a similar increase in acrosomal responsiveness to LPC (Fig. 1C).

We also evaluated a possible increase in tyrosine phosphorylation of sperm proteins, because it has been temporally correlated with capacitation [2], [32]. The addition of 1.4 nM Met-AEA or 1 nM AEA to sp-TALP medium displayed an increase in the phosphorylation of tyrosine residues of a group of proteins in bovine spermatozoa (Fig. 1D), as observed with heparin used as positive control. In support of the results of Western immunoblotting, immunocytochemistry assays revealed a strong signal on the sperm head when spermatozoa were capacitated for 45 min in the presence of 1.4 nM Met-AEA or heparin as compared with the control (Fig. 1E).

So far, our results indicate that nanomolar concentrations of AEA or Met-AEA induced bull sperm capacitation. Given that AEA and Met-AEA produced similar effects, the following experiments were performed only with Met-AEA.

**Figure 2:**
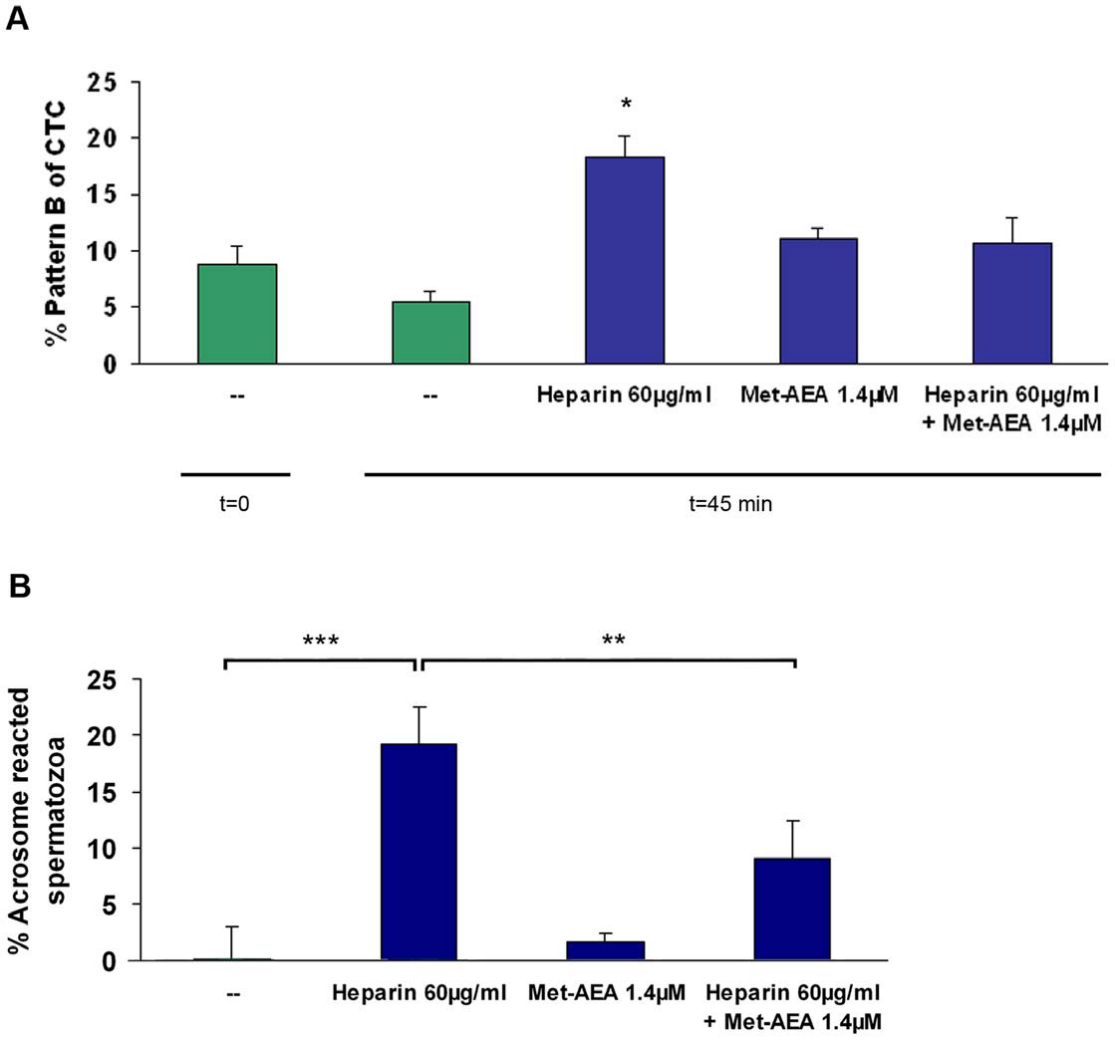
Effect of micromolar concentration of Met-AEA on bull heparin-induced sperm capacitation. Spermatozoa were incubated for 45 min at 38.5°C in sp-TALP medium with heparin, Met-AEA (1.4 µM) or Heparin + Met-AEA (1.4 µM). Bars indicate the percentage of capacitated spermatozoa (A: % pattern B of CTC; B: % acrosome reacted spermatozoa). Data are expressed as mean ± SEM. **A**: Assessment of sperm capacitation by CTC assay. (—) t = 0 and t = 45, sp-TALP at 0 and 45 min (control) incubation respectively (n = 4). *p<0.05 vs control and Heparin (60 µg/ml) + Met-AEA (1.4 µM). **B**: Assessment of sperm capacitation by acrosome reaction evaluated by PSA-FITC; (—) t = 45 (control) (n = 4). ***p<0.001 vs control; **p<0.01 vs Heparin (60 µg/ml) + Met-AEA (1.4 µM).

We also investigated whether bull sperm capacitation induced by heparin (60 µg/ml) is modified by the presence of micromolar concentrations of Met-AEA. We found, by CTC and LPC-induced acrosome reaction, that micromolar concentrations of Met-AEA inhibited heparin-induced sperm capacitation (Figures 2A, 2B) but those concentrations alone did not exert any inhibitory effect (Figures 2A, 2B).

### Expression and localization of TRPV1 in bovine spermatozoa

**Figure 3:**
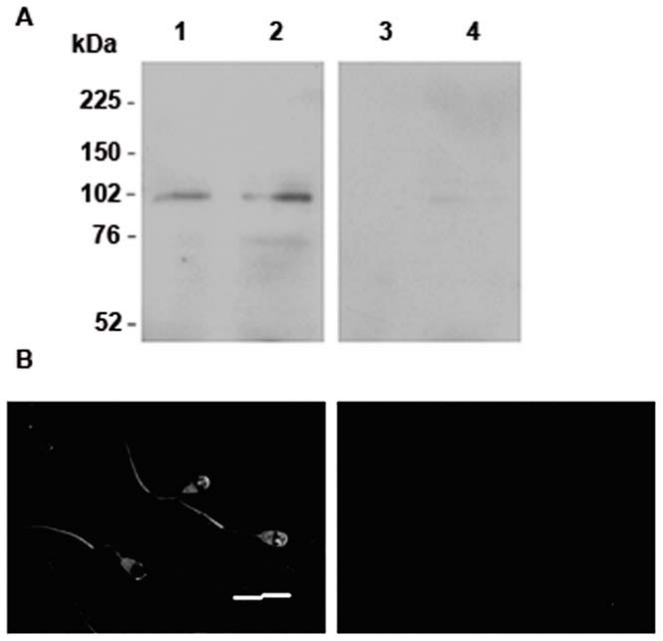
Expression and localization of TRPV1 in bovine spermatozoa. **A**: Sperm proteins were extracted and subjected to SDS-PAGE immunoblotting with a specific antibody against TRPV1. Lanes 1–2: antiTRPV1, lanes 3–4: antiTRPV1 plus blocking peptide, (n = 3). **B**: Immunolocalization of TRPV1; left panel: spermatozoa incubated with antiTRPV1; right panel: spermatozoa incubated with IgG fractions from non-immunized rabbits at the same concentration that the primary antibody (n = 3); Scale bar: 20 µm (Magnification, X600).

Since our results suggested that AEA induces sperm capacitation, we then investigated its possible activation pathway. We have previously found that bull spermatozoa express CB1 receptors [20]. In the present work, we studied the expression of TRPV1 in spermatozoa since this receptor is also a divalent ion channel and it is known that calcium is involved in sperm capacitation [13], [30]. Western blot analysis showed a single immunoreactive band of the molecular size expected for TRPV1 (∼100 kDa); this band was absent when the blocking peptide was added (Fig. 3A). Also, immunocytochemical analysis showed TRPV1 localization predominantly on the tail, the apical region of the acrosome and the post-acrosomal region in sperm head (Fig. 3B). Staining was specific because spermatozoa incubated with IgG fractions from non-immunized rabbits (Fig. 3B) or without the primary antibody (data not shown) did not show any signal.

### Effects of CB1, CB2 and/or TRPV1 antagonist in AEA-induced sperm capacitation

**Figure 4:**
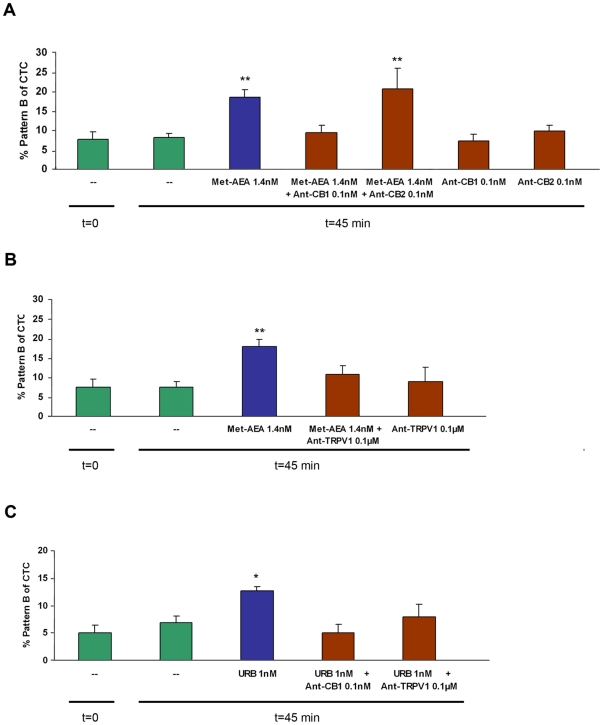
Evaluation of CB1, CB2 and TRPV1 participation in bull sperm capacitation. Spermatozoa were incubated for 45 min at 38.5°C with different treatments. Bars indicate the percentage of capacitated spermatozoa (pattern B) determined by CTC. Data are expressed as mean ± SEM. **A**: sp-TALP (control), Met-AEA (1.4 nM), Met-AEA plus Ant-CB1 (CB1 antagonist SR141716A; 0.1 nM), Met-AEA plus Ant-CB2 (CB2 antagonist SR144528); **p<0.01 Met-AEA vs. control and Met-AEA plus Ant-CB1 (n = 6). **p<0.01 Met-AEA plus Ant-CB2 vs. control and Ant-CB2 (n = 4). **B**: sp-TALP (control), Met-AEA (1.4 nM) or Met-AEA plus Ant-TRPV1 (TRPV1 antagonist capsazepine; 0.1 µM); **p<0.01 Met-AEA vs. control and Met-AEA plus Ant-TRPV1 (n = 6). **C**: sp-TALP (control), URB597 (FAAH inhibitor; 1 nM), URB597 plus Ant-CB1 or URB597 plus Ant-TRPV1; *p<0.05 URB597 vs. control, URB597 plus Ant-CB1 and URB597 plus Ant-TRPV1 (n = 5). (—) t = 0 and t = 45, sp-TALP at 0 and 45 min (control) incubation respectively.

To determine the possible AEA activation pathway, sperm capacitation was performed with SR141716A (a CB1 antagonist: [33]), SR144528 (a CB2 antagonist: [34]) or capsazepine (a TRPV1 antagonist: [18]). The presence of SR141716A inhibited Met-AEA-induced capacitation, but the antagonist SR144528 did not reverse the effect of the cannabinoid agonist (Figure 4A).

As shown in Figure 4B, capsazepine also inhibited the effect of Met-AEA. The incubation with SR141716, SR144528 or capsazepine alone did not have any effect.

Since the CB2 antagonist did not reverse the Met-AEA effect, the following experiments were performed only with CB1 and TRPV1 antagonists.

To explore whether an increase of endogenous AEA might induce sperm capacitation, spermatozoa were incubated with URB597, a selective FAAH inhibitor [35]. Results indicate that the percentage of capacitated spermatozoa increased when cells were treated with 1nM URB597 as compared to the control (Fig. 4C). The pre-incubation with CB1 or TRPV1 antagonists reversed the effect of URB597 (Fig. 4C).

### Evaluation of the AEA pathway activation in the heparin-induced sperm capacitation

**Figure 5:**
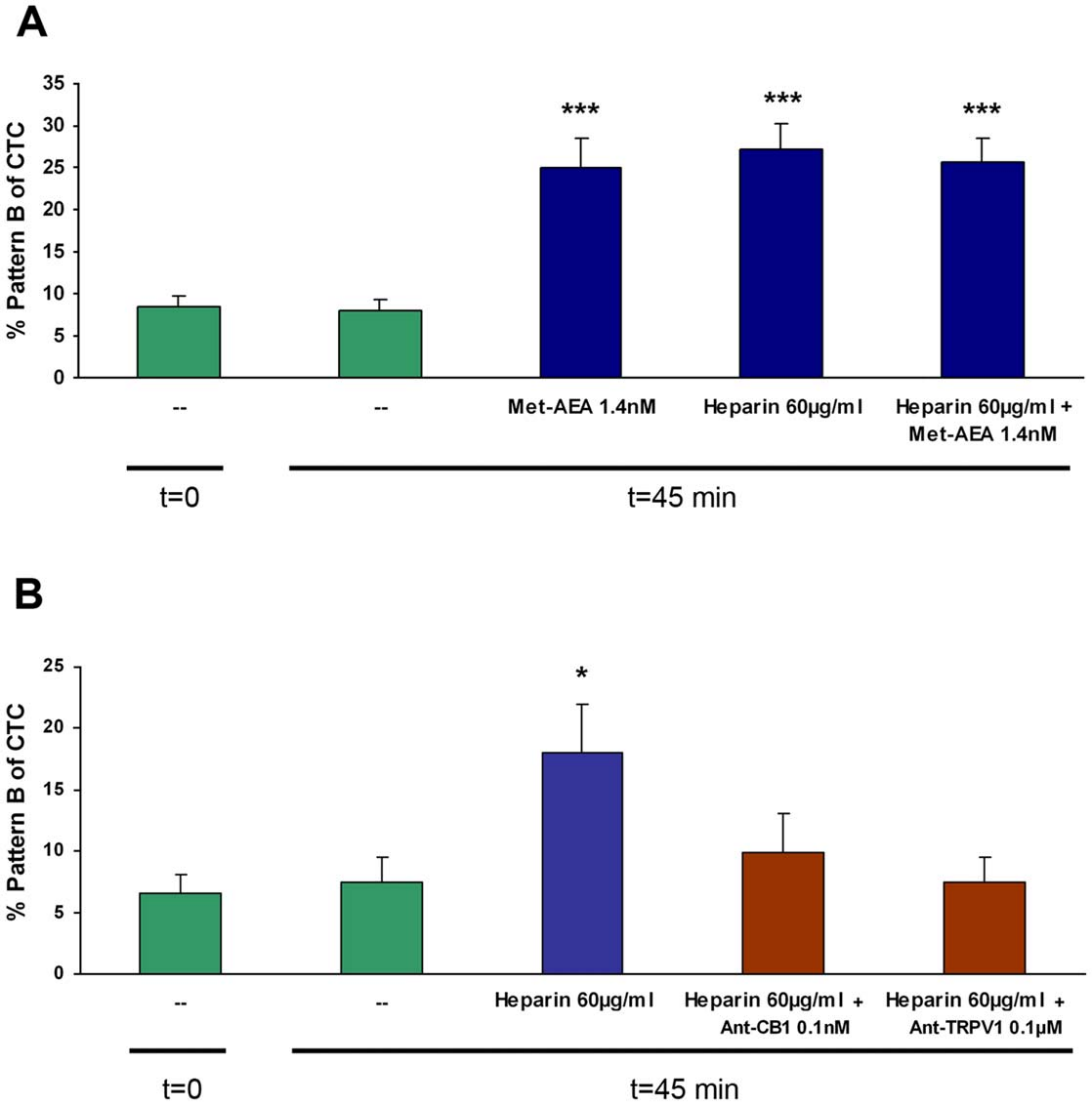
Effect of nanomolar concentration of Met-AEA and heparin on bull sperm capacitation. Spermatozoa were incubated for 45 min at 38.5°C with different treatments. Bars indicate the percentage of capacitated spermatozoa (pattern B) determined by CTC. (t = 0; t = 45: controls). Data are expressed as mean ± SEM. **A**: Met-AEA, Heparin or Met-AEA plus Heparin. ***p<0.001 vs. control (n = 6). **B**: Heparin, Heparin plus Ant-CB1 or Heparin plus Ant-TRPV1. *p<0.05 Heparin vs. control, Ant-CB1 and Ant-TRPV1 (n = 4).

To investigate whether AEA is involved in bovine spermatozoa heparin-capacitation, spermatozoa were incubated with heparin, Met-AEA or heparin + Met-AEA. The percentage of capacitated spermatozoa (Pattern B) was similar in all treatments (Fig. 5A). These results were supported by LPC-induced acrosome reaction (Control: 1.20±0.92%; Met-AEA: 8.95±1.28%; heparin: 13.51±2.11%; Met-AEA + heparin: 9.94±0.54%; n = 7, p<0.05).

The results of sperm capacitation suggested that AEA and heparin could share the same activation pathway. We performed an experiment to study whether heparin could promote sperm capacitation by activating the AEA signaling pathway. The results indicated that the ability of heparin to induce sperm capacitation was inhibited by the presence of TRPV1 or CB1 antagonists (Fig. 5B).

Previously, we demonstrated that bull spermatozoa express FAAH [20]. Then, FAAH activity was measured in heparin-capacitated spermatozoa. After 45 min incubation, a decrease in FAAH activity was observed as compared with that found before the incubation (Control: 104.57±4.78; Heparin: 74.67±7.89 nmol AA/h/mg protein; n = 5, p<0.01).

## Discussion

In this work, we demonstrated, for the first time, that physiological concentrations of AEA were capable of inducing bovine sperm capacitation. Interestingly, both AEA and Met-AEA (a non-hydrolysable AEA analogue) elicited the same response as that obtained with heparin, a known capacitating agent in bovines. Percentages of *in vitro* capacitation obtained in our experiments with cryopreserved spermatozoa are similar to those found by other authors with different capacitation inducers [36]–[37].

The significant role of AEA on sperm capacitation was also reflected on the increase of tyrosine phosphorylation levels of sperm proteins found in treated gametes.

In addition, our results show that spermatozoa that had been incubated for 45 min in sp-TALP with AEA or Met-AEA increased their ability to undergo the acrosome reaction stimulated with the fusogenic lipid LPC, supporting again that AEA in bull spermatozoa could behave as a capacitating agent.

The participation of the endocannabinoid system in the mammals' reproductive functions has been widely demonstrated [20], [23], [29], [38]–[39]. Previous reports indicate that endocannabinoid signaling is implicated in the reproductive system of the male, attenuating different aspects involved in sperm maturation and the acquisition of fertilizing capacity [22]–[23], [40]. Most of the *in vitro* experiments conducted with endocannabinoids and spermatozoa suggest that AEA is a molecule that diminishes sperm function and also that high (micromolar) concentrations of AEA, Met-AEA or Δ9-tetrahydrocannabinol inhibit motility, capacitation and acrosome reaction of human and boar spermatozoa [21]–[23]. In addition, the genetic loss of FAAH results in elevated levels of AEA in the male reproductive system, leading to compromised fertilizing capacity of sperm in mice [41].

According to those reports, we also demonstrated that micromolar concentrations of Met-AEA blocked heparin-induced sperm capacitation. However, the incubation of bovine sperm with micromolar concentrations of Met-AEA alone did not have any inhibitory effect on sperm capacitation.

All these results suggest that the AEA levels may be finely regulated in the male gamete.

We have previously found that AEA is involved in sperm-oviduct interaction through CB1 receptors [20], suggesting functional roles for AEA-signaling in regulating sperm capacitation and fertilizing potential. In this work our observations suggest that bovine sperm capacitation is stimulated by endocannabinoids via CB1 activation because AEA effects were fully antagonized by the CB1 antagonist SR141716A. However, CB2 antagonist did not reverse the effect of Met-AEA, suggesting that CB2 is not involved in the induction of bull sperm capacitation elicited by AEA. Since sperm capacitation is one of the possible causes that promotes sperm releasing from bovine oviductal epithelium, these results are consistent with those found previously by Gervasi *et al.* [20], indicating that AEA at nanomolar concentrations promotes sperm releasing from bovine oviductal epithelium, by CB1 and TRPV1 activation but not through CB2.

The endocannabinoid system may play a role in the modulation of TRPV1 activation [18]. Although that TRPV1 has been found previously in others mammalian spermatozoa [21]–[22], in this work we demonstrated, for the first time, that this receptor is also expressed in bull spermatozoa. Immunofluorescence analysis of bull spermatozoa revealed TRPV1 staining over the post-acrosomal region and on the tail of bull spermatozoa corroborated the previous findings [21]–[22], although we also detected immunoreactivity in the acrosome (apical region). The functionality of TRPV1 was observed when Met-AEA-induced capacitation was inhibited by capsazepine, suggesting that TRPV1 activation by AEA is also involved in this process.

The efficacy of AEA as a TRPV1 agonist is influenced by a succession of factors including receptor reserve, phosphorylation, metabolism and uptake, and CB1 receptor activation [18]. Cross-talk between TRPV1 and CB1 receptors co-expressed in the same cells might also include reciprocal influences on receptor activation. In fact, CB1, TRPV1 and calcium ions seem to be in the same signaling pathway in some biological systems [18]. Thus, we speculate that in our model an increase in AEA levels could activate sperm CB1 and/or TRPV1, leading to increased intracellular calcium concentrations required for sperm capacitation.

Heparin and other molecules, such as nitric oxide and thiol-reducing agents, are able to trigger sperm capacitation through different pathways or sharing mechanisms [15], [42]–[43]. Heparin-like glycosaminoglycans are present in the bovine oviductal fluid, and changes in their concentration and capacitating activities are maximal at estrous [14]–[15]. Our results suggest that heparin and AEA may share the same molecular pathways, because 1) the percentage of capacitated sperm incubated with both molecules was similar to those percentages found when sperm was incubated with AEA or heparin alone and 2) the heparin-induced capacitation was inhibited by CB1 or TRPV1 antagonists. Furthermore, FAAH activity decreased in spermatozoa incubated with heparin, suggesting an endogenous increase of AEA as this enzyme is responsible for the degradation of the endocannabinoid.

A reduction in FAAH activity after incubation under capacitating conditions concomitantly with an increase in AEA levels in capacitated boar spermatozoa was previously reported [22]. According to these authors an increase of intracellular AEA during sperm capacitation prevents spontaneous acrosome reaction and maximizes the responsiveness to physiological acrosome reaction inducers through TRPV1. Other authors also provided evidences that the AEA-binding TRPV1 receptor could be involved in human sperm functions [21]. Thus, an increase of AEA could be fulfilling an important role by promoting mechanisms involved in the acquisition of the sperm fertilizing ability.

Blocking AEA degradation by URB97 (a potent FAAH inhibitor) elicited sperm capacitation and the pre-incubation with CB1 or TRPV1 antagonists reversed this effect. These data support our previous results, suggesting that an increase of endogenous AEA also induces sperm capacitation. These evidences indicate that in bull spermatozoa, the sperm capacitation may be normally regulated by an endogenous AEA tone.

The mechanisms underlying AEA participation in sperm capacitation have not yet been elucidated. The activation of CB1 in various cell types has been coupled to ion channel modulation [44]–[45]. Previous data indicate that AEA, through CB1, induces rapid membrane hyperpolarization by activating potassium effluxes from sperm cytoplasm [46]. Additionally, AEA might produce an increase in sperm calcium concentration via TRPV1.

Physiologically, sperm capacitation occurs within the female genital tract and it has been demonstrated that sperm cells leaving seminal plasma and reaching the uterus and the oviduct swim in an external milieu presenting an AEA gradient [47]. Anandamide from two potential sources could be involved in the regulation of sperm capacitation: 1) AEA produced by sperm [22] and 2) AEA derived from somatic cells lining the oviduct [38], [47]. Previous results indicate that AEA, at nanomolar but not lower concentrations, induces sperm release from bovine oviductal epithelium [20]. Based on these results and previous reports that nanomolar levels of AEA are found in human mid-cycle oviductal fluids [27], we speculate that oviductal AEA secretion during the peri-ovulatory (oestrogenic influence) period might be one of the signals that participate in the fertilization process promoting sperm capacitation and thus enhancing the ability of spermatozoa to be released from oviductal reservoirs. Also, AEA as a second messenger might mediate the effect of other molecules, such as heparin-like glycosaminoglycans, present in the oviductal fluid and involved in sperm function signaling pathways. Therefore we consider that the participation of AEA in regulating sperm capacitation could involve both exogenous AEA (from the oviduct) and/or endogenous AEA (from the spermatozoa).

In summary, we demonstrated for the first time that AEA is able to capacitate bull spermatozoa through CB1 and/or TRPV1 activation and that it could be involved in the heparin signaling pathway. These findings provide novel information about the involvement of the endocannabinoid system in regulating mammalian sperm capacitation. In addition, knowledge of new molecular pathways in the regulation of sperm function could be useful in the search for better diagnosis and treatment of male infertility.

## Materials and Methods

### Chemicals

R(+)-methanandamide, anandamide, luminol, and p-coumaric acid, bovine serum albumin (BSA), heparin, chlortetracycline (CTC), Pissum-sativum (PSA-FITC), capsazepine, Hoescht 33258 and L-α-lysophosphatidylcholine (LPC) were purchased from Sigma Chemicals (St. Louis, MI, USA). URB597 were obtained from Cayman. PVDF and nitrocellulose membranes were obtained from BioRAD (Hercules, CA, USA). Glass wool to perform columns was obtained from Micro-Fibre Manville (Denver, CO, USA). SR141716A (N-piperidino-5-(4-chlorophenyl)-1-(2,4-dichlorophenyl)-4-methyl-3-pyrazole carboxamide and SR144528 (N-[1(S)-endo-1,3,3-trimethyl-bicyclo[2.2.1]heptan-2-yl]-5-(4-chloro-3-methylphenyl)-1-(4-methyl-benzyl)-pyrazole-3-carboxamide) were kind gift from Sanofi-Aventis Recherche (Montpellier, France). TRPV1 antibodies were purchased from Santa Cruz Biotechnology (peptide fragment: (C)EDAEVFKDSMVPGEK corresponding to residues 824-838, C-terminus; Santa Cruz, CA, USA) and Alomone Labs (peptide fragment: amino acids corresponding to residues1-130, N-terminus; Jerusalem, Israel). Phosphotyrosine antibodies were purchased from Upstate Technologies-Millipore (clone 4G10, Immunogen: phosphotyramine-KLH; MA, USA) and Affinity BioReagent (Rockford, IL, USA). Anti-mouse IgG conjugates HRP or CY3 were obtained from Jackson (ImmunoResearch Laboratories, Baltimore, PA, USA) and Alexa-Fluor555 goat anti-rabbit IgG was from Molecular Probes (Invitrogen, Carlsbad, CA, USA). All other chemicals were of analytical grade.

### Culture media

Sperm handling was performed with Tyrode bicarbonate buffered medium without BSA (BSA-free sp-TALP) and capacitation experiments with sp-TALP supplemented with 0.3% BSA [12].

### Sperm preparation

Frozen bovine semen from six bulls (15×10^6^ spermatozoa/0.5 ml straw), obtained from CIAVT (Artificial Insemination Cooperative Venado Tuerto, Santa Fé-Argentina), was used. Straws were thawed in a water bath (37°C for 30 s). Spermatozoa were subjected to sperm selection using glass wool columns [48] and washed by centrifugation for 5 min at 800 g with BSA-free sp-TALP. Pellets were assessed for sperm concentration and motility using a haemocytometer mounted on a microscope stage heated at 38°C. Samples presenting 70% average of progressive motility were considered suitable for experiments.

### SDS-PAGE and immunoblotting

Sperm proteins from 3×10^6^ spermatozoa were obtained as described by Visconti *et al.* [2] and then separated in 10% polyacrylamide SDS-PAGE gels [49].

### TRPV1 detection

Samples were transferred to nitrocellulose membranes according to the method of Towbin *et al.* [50] at 30 V (constant) over night. TRPV1 protein expression was determined by Western immunoblotting analysis using specific antibodies. Membrane non-specific binding sites were blocked (5% w/v dried fat milk, 1 h at room temperature (RT)) and incubated with primary TRPV1 (1∶200 v/v in PBS) antibody followed by incubations with goat anti-rabbit HRP-conjugated IgG. Immunoreactive specificity was assessed by incubation with blocking peptide.

### Phosphotyrosine detection

Electrophoretic transfer of proteins to PVDF membranes in all experiments was carried out at 30 V (constant) for 6 h at 4°C. Immunodetection of proteins on membranes was performed at RT as described previously [51] using a monoclonal antibody against phosphotyrosine (1∶1000 PBS; clone 4G10; [32]). As internal control, all membranes were subsequently stripped (β-mercaptoethanol 0.1 M, 10% SDS in buffer TRIS 62.5 mM for 15 min at 50°C) and reprobed with anti-β tubulin antibody.

Protein bands were visualized using chemiluminescence detection reagents and exposed to Kodak X-OMAT films (Sigma Co).

### Immunocytochemistry

Spermatozoa were fixed (5 min, RT, 0.2% w/v paraformaldehyde), immobilized on slides and permeabilized with cold methanol.

#### TRPV1 localization

Non-specific binding sites were blocked (60 min, 10% v/v normal goat serum) and slices were treated with primary TRPV1 antibody (1∶50). Afterwards, samples were incubated with Alexa555-conjugated goat anti-rabbit IgG (1∶2000).

#### Phosphotyrosine localization

Non-specific binding sites were blocked (60 min, 40 mg/ml BSA–PBS) and spermatozoa were incubated with phosphotyrosine antibody (1∶50). Then slices were incubated with anti-mouse CY3-conjugate (1∶1000).

Specificity of the immunodetection was assessed by omitting the first antibody (TRPV1 and phosphotyrosine) or by the replacement of specific primary antibody with IgG fractions from non-immunized rabbits at the same concentration (TRPV1). Sperm cells were mounted and examined under a fluorescence microscope (Olympus IMT2, Japan) coupled to a digital camera and examined under a confocal laser imaging system (Nikon C1; Plan Apo 60/0.95, Japan).

### *In vitro* spermatozoa capacitation

Spermatozoa were washed as described above and incubated at 38.5°C, 5% CO_2_ atmosphere. Ten to 15×10^6^ cells/ml were placed in 0.3% BSA/sp-TALP for 45 min [37], [52] with increasing concentration of Met-AEA (0.14 nM to 1.4 µM), AEA (1 nM), heparin (60 µg/ml), SR141716A (0.1 nM), SR144528 (0.1 nM) Capsazepine (0.1 µM) and URB597 (1 nM). Capacitation was assessed as described below.

### Chlortetracycline (CTC) assay

CTC fluorescence assays were carried out as described previously by (Ward & Storey [53]) to assess sperm capacitation. This was determined by the ability of spermatozoa to display CTC fluorescence pattern B, indicative of capacitating status [53]–[54]. Pattern B evaluation was performed on initial sperm suspensions (t = 0) and after 45 min (t = 45) incubation under the conditions mentioned above.

### Induction of acrosome reaction and evaluation of acrosomal status

The induction of acrosome reaction was performed as described previously [32] with modifications. Spermatozoa were incubated with the agents for 45 min and then divided in two aliquots of 200 µl; one of them was incubated with LPC (100 µg/ml) and the other without it for 15 min at 38.5°C. To assess viability and acrosome reaction spermatozoa were incubated with H258 (2 µg/ml) for 5 min, fixed (1% w/v paraformaldehyde) for 8 min at RT and washed with PBS. An aliquot was air-dried onto slides and permeabilized in methanol for 10 min at 4°C. Slides were incubated with 50 µg/ml PSA-FITC for 60 min at RT [55]–[56]. At least 200 stained cells/treatment were scored in an epifluorescence microscope. The percentage of capacitated spermatozoa was represented by the difference between percentages of viable-acrosome-reacted spermatozoa in LPC-treated and in non-LPC-treated samples.

### Measurement of FAAH activity

FAAH activity was assayed as described by [57]. The hydrolysed (^3^H)-AA (arachidonic acid) was resolved in the organic layer of a solvent system of ethyl acetate:hexane:acetic acid:distilled water (100∶50∶20∶100 v/v) mixture. The plate was exposed to iodine to identify the AA areas. The distribution of radioactivity on the plate was counted in a scintillation counter by scraping off the corresponding spots detected in the plate. The area of each radioactive peak corresponding to AA was calculated and expressed as a percentage of the total radioactivity of the plates. Enzyme activity is reported as nmol (^3^H)-AA/mg protein/h. Protein concentration was determined by the Bradford assay [58]. The optimal reaction conditions were previously determined (data not shown).

### Statistical analysis

Data were analyzed by GLM procedures of one-way ANOVA (Statistica 6.0 software, StatSoft Inc., Tulsa, USA, 1984–2001). Raw data were analyzed by Cochran, Hartley and Bartlett test and GLM procedures were applied in all variance analyses. Data that did not fulfill assumptions were normalized by the arcsine transformation. Pairwise comparisons of means were made with Tukey or Fisher honestly significant differences. Results are expressed as mean±s.e.m of at least three independent determinations. Differences were considered significant when p<0.05 or less.
